# Supplementary material for: Histone exchange sensors reveal variant specific dynamics in mouse embryonic stem cells
Source: Nat Commun. 2023 Jun 26;14:3791. doi: 10.1038/s41467-023-39477-3 (PMC10293259; doi:10.1038/s41467-023-39477-3)

Supplementary Fig. 1

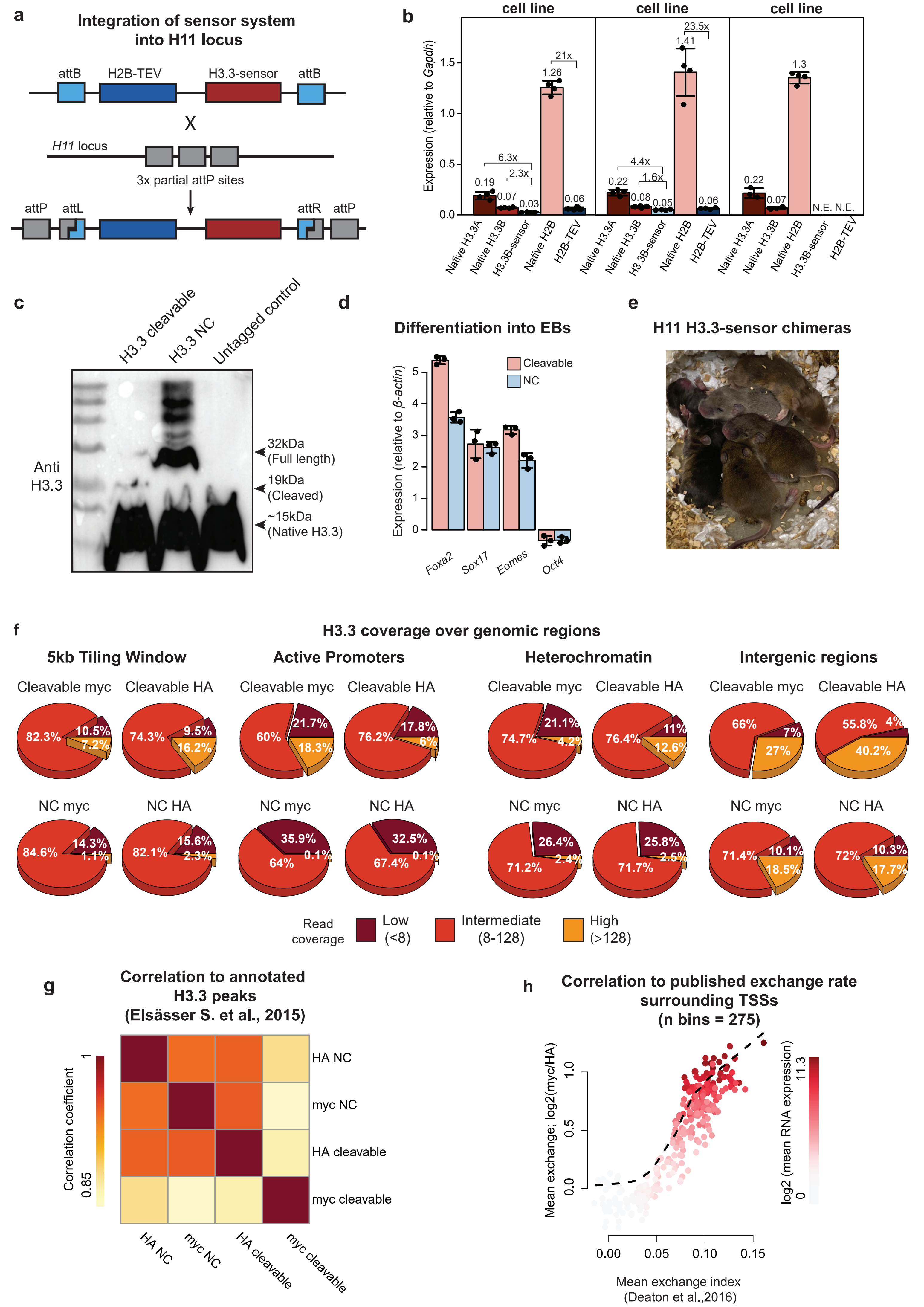

Supplementary Fig. 2

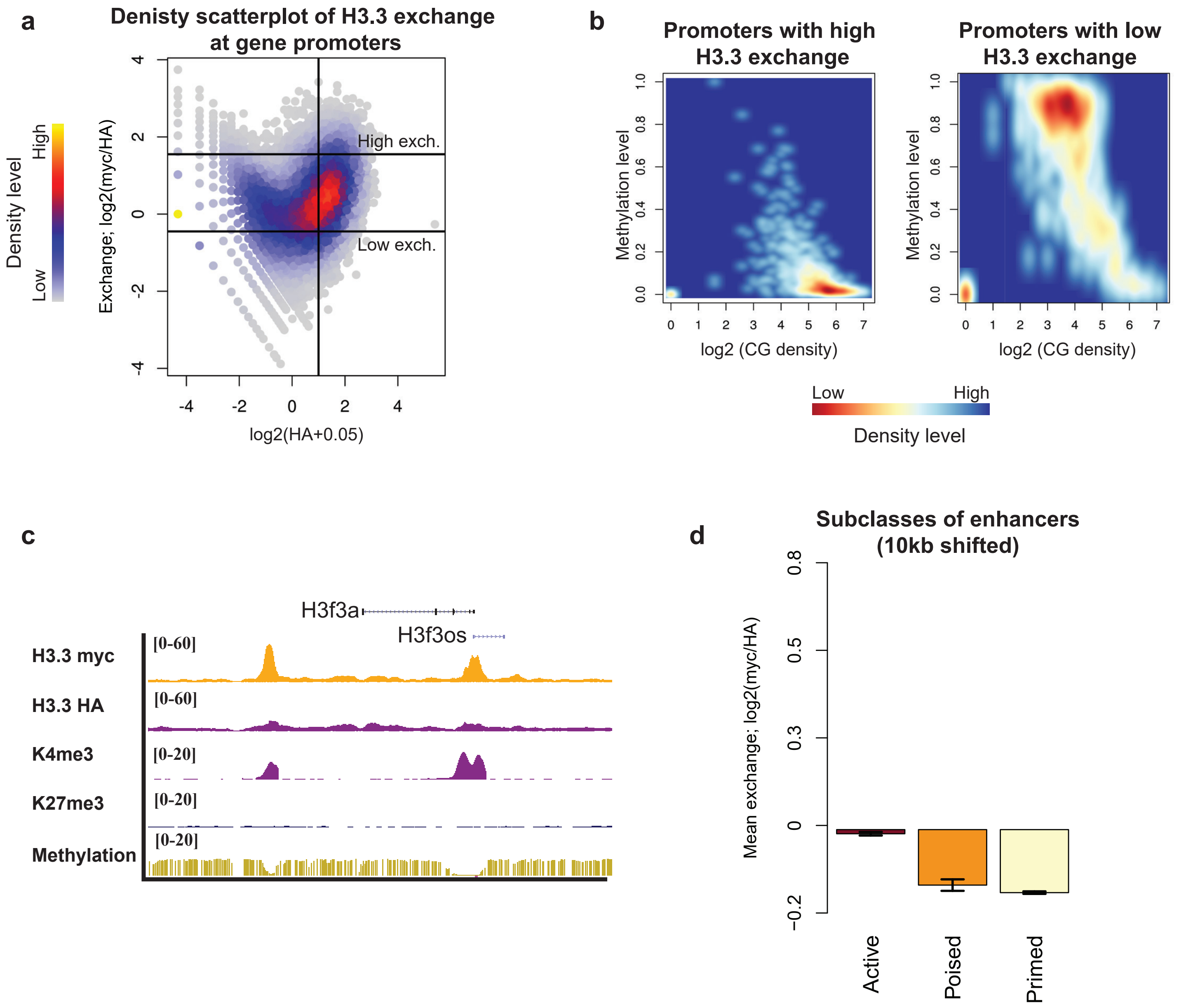

Supplementary Fig. 3

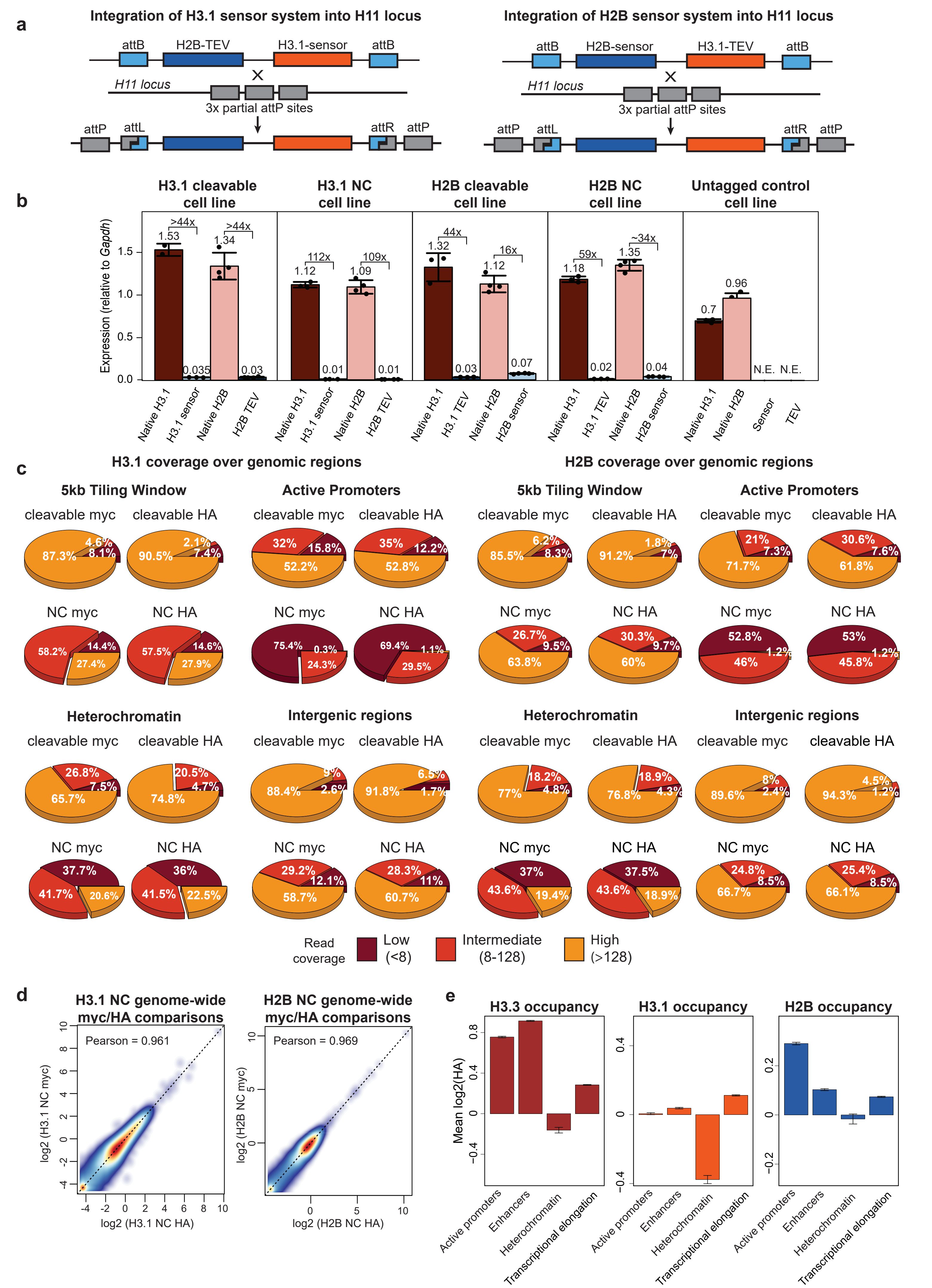

Supplementary Fig. 4

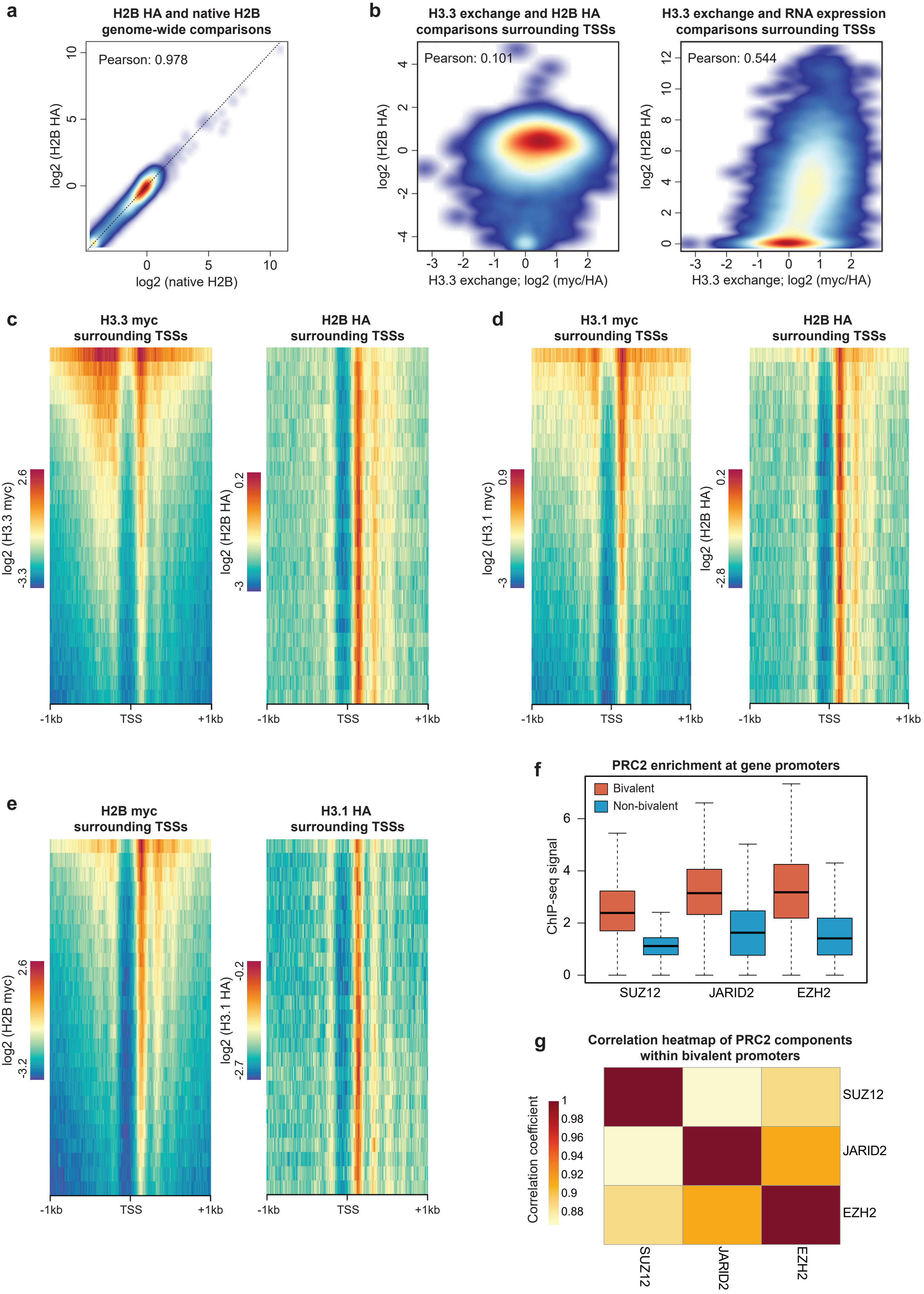

Supplementary Fig. 5

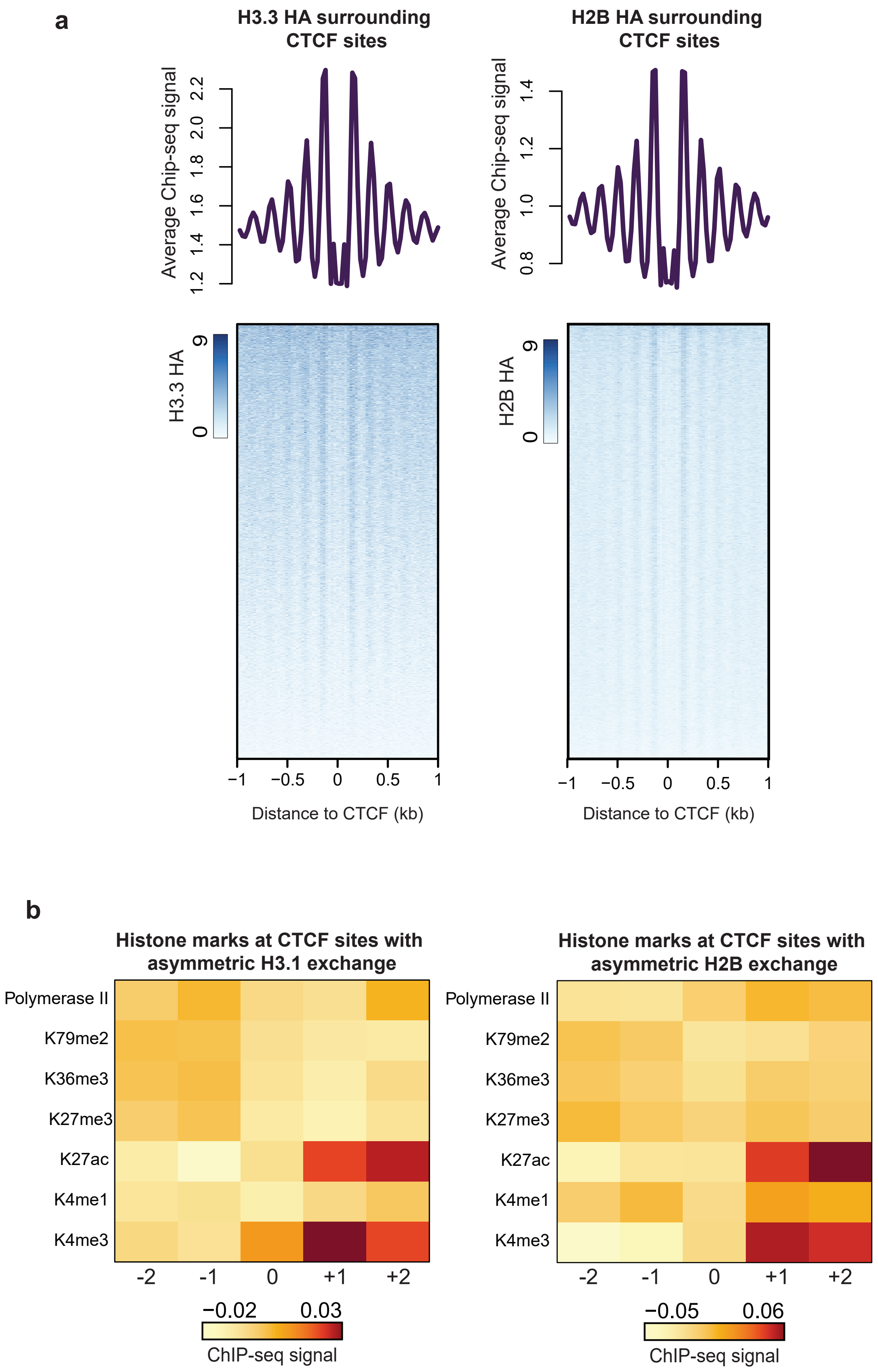

Supplementary Fig. 6

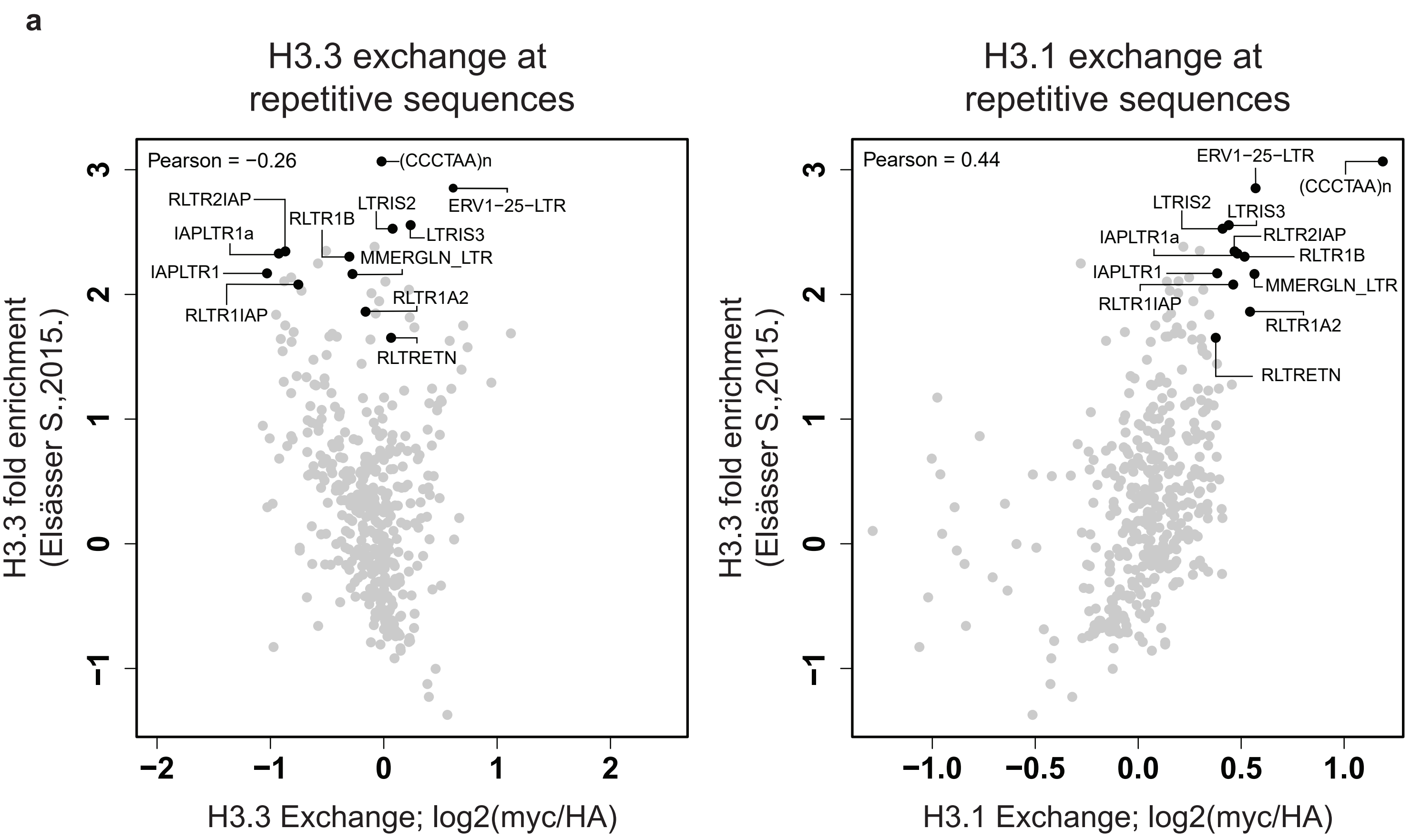

Supplementary Fig. 7

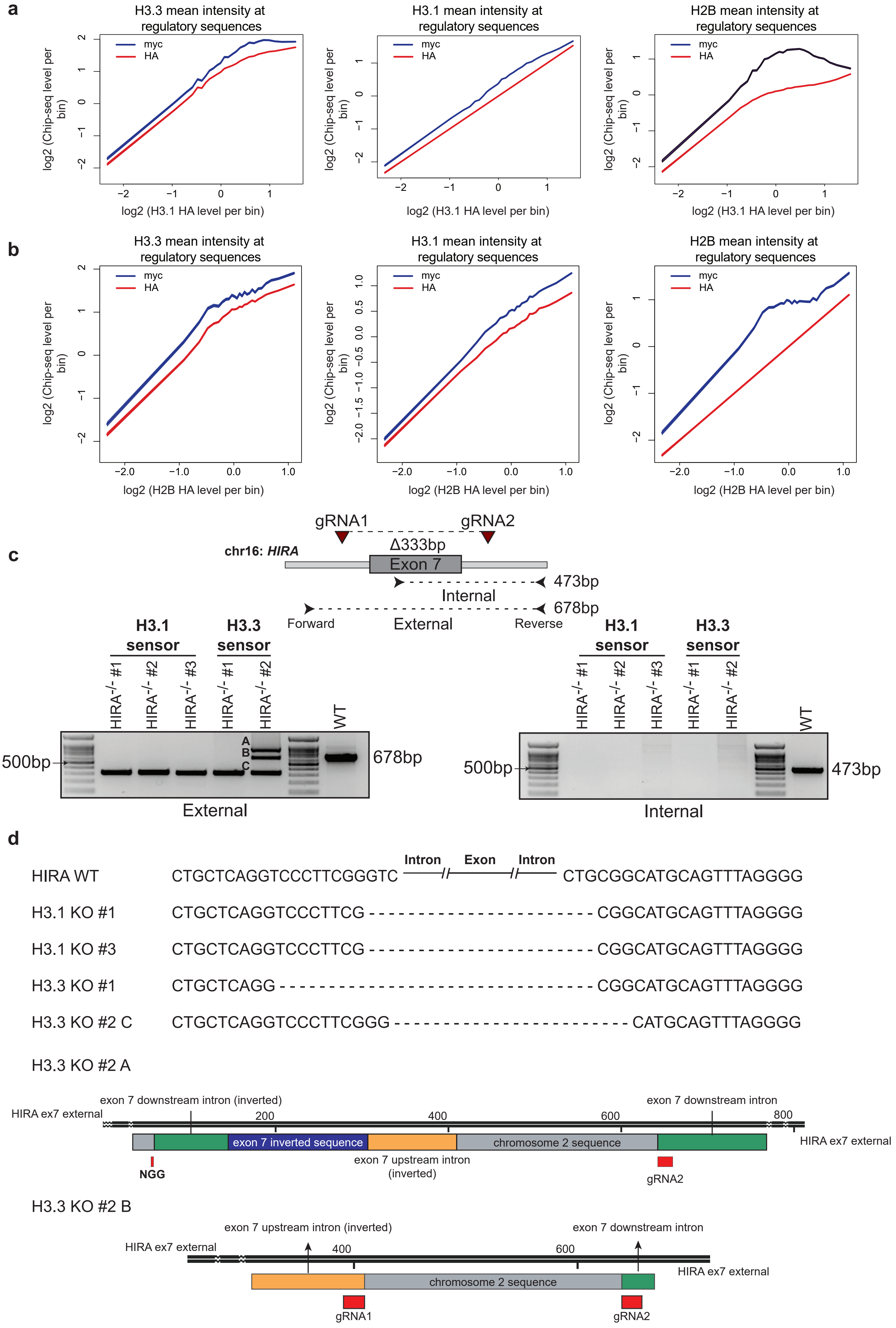

Supplementary Fig. 8

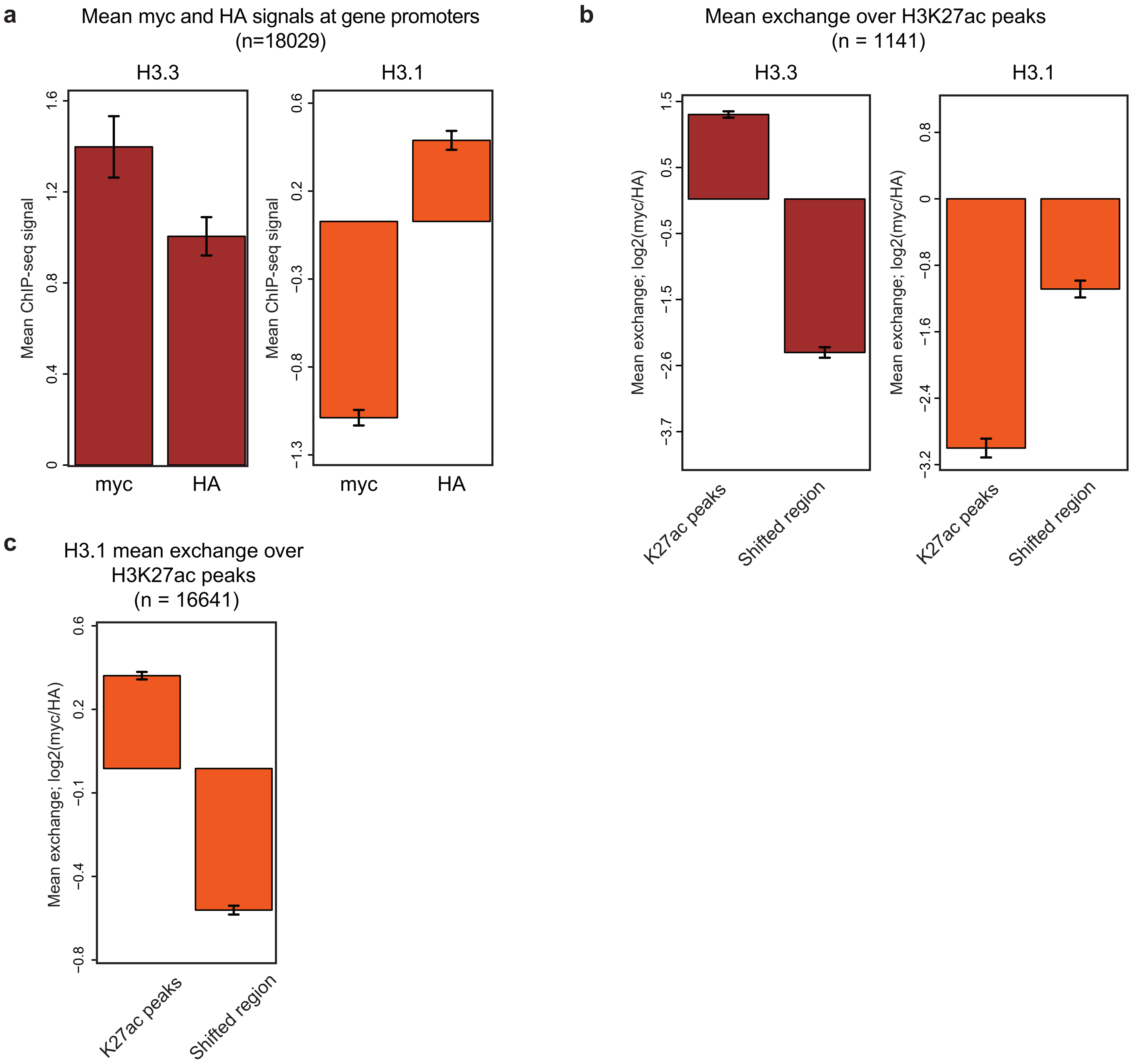

Supplementary data figure legends

Supplementary Fig. 1: Generation of reporter cell line of H3.3 exchange and validation of the system

a) Schematic representation of recombination mediated integration of transgene into *H11* locus. DNA integration cassette of donor plasmid is flanked by two attB attachment sites. H11 locus contains three partial attP sequences. Site-specific recombination between attB and one of attP sites results in formation of hybrid attL and attR sites and insertion of DNA cassette with tagged histones.

b) RT-qPCR relative expression levels of endogenous H3.3A and H3.3B genes as compared to H3.3-HA and native H2B as compared to H2B-TEV signals for cleavable and NC cell lines, respectively. Expressions are represented relative to *Gapdh* (log2). Data are presented as mean values ± SD. Error bars denoting SD of three or four technical replicates. N.E. standing for non-expressed. Indicated are fold differences between native and tagged histones.

c) Western blot analysis on nuclei extract with H3.3-specific antibody. Tagged H3.3 variants are distinguished from their native counterparts by size differences. Blots are representative of two independent experiments.

d) RT-qPCR expression changes of pluripotency and differentiation marker genes in cleavable and NC cell lines subjected to spontaneous differentiation into embryoid bodies (EBs). Gene expressions are represented relative to beta-actin expression levels and normalized to expression in naïve mESCs. Data are presented as mean values ± SD. Error bars denoting SD of three technical replicates. Experiment was performed one time.

e) Representative images of postnatal chimeric mice bearing H3.3-sensor system generated by microinjection of modified mESC into blastocysts.

f) Distribution of read sums (log2) per sample within indicated genomic region. A 5-kb sized window was tiled through the genome, whereby coordinates of active promoters, heterochromatin and intergenic regions were obtained from https://github.com/guifengwei/ChromHMM_mESC_mm10. Reads are categorized into three groups based on coverage: high, intermediate and low coverage. Read sum per group was more than 128, 8-128 and less than 8, respectively.

g) Pearson correlation matrix of myc and HA tags between cleavable and NC samples within H3.3 peaks.

h) Represented are 275 gene expression-ranked bins. Mean exchange level of each bin is plotted against mean exchange index of published dataset from[26]. Colors indicate mean expression level per bin (log2). Source data are provided as a Source Data file.

Supplementary Fig. 2: Association between H3.3 exchange and epigenetic landscape of gene promoters and regulatory elements.

a) Density scatterplot of H3.3 exchange rate and H3.3 occupancy within gene promoters (n=31932) used to define promoters of high and low exchange (see Methods).

b) Density plot showing correlation between methylation and CpG density level for two gene categories classified by exchange levels of H3.3 within gene promoters. Number of promoters with high H3.3 exchange = 1303; Number of promoters with low H3.3 exchange = 964;

c) Representative genomic region from UCSC illustrating myc and HA signal within transcriptionally active genes.

d) H3.3 mean exchange over control regions shifted 10kb downstream of annotated enhancer classes. Data are represented as mean ± SEM. Number of regions are 12142 (active), 19723 (primed), and 1015 (poised) enhancers. Source data are provided as a Source Data file.

Supplementary Fig. 3: Construction of cell lines carrying H3.1 and H2B sensor systems

a) Schematic representation of site-specific recombination targeting of H3.1-tagged and H2B-tagged transgenes into *H11* locus.

b) RT-qPCR relative expression levels of endogenous H3.1 and H2B genes as compared to HA or TEV signal of H3.1 and H2B modified variants for cleavable and NC cell lines. Expressions are represented relative to *Gapdh* (log2). Data are presented as mean values ± SD. Error bars denoting SD of three or four technical replicates. N.E. standing for non-expressed. Indicated are fold differences between native and tagged histones.

c) Read coverages for 5kb tiling windows and respective genomic regions defined as in Supplementary Fig. 1f. Sum of reads are categorized into one of three groups (high, intermediate and low coverage).

d) Genome-wide correlation of myc and HA counts for NC forms of sensor. Read counts are calculated within 5kb tiling windows. Number of analyzed windows for H3.1 NC sample was 544473, and 544479 for H2B NC sample. Indicated are Pearson correlation coefficients for all windows. Dashed is 1:1 reference line.

e) Barplot representation of H3.3, H3.1 and H2B mean occupancies within the same genomic regions as in Fig. 2c. Within each region, mean occupancy (defined by log2 HA) was normalized by subtracting to mean occupancy of 20kb shifted control region. Data are represented as mean ± SEM. Number of regions are 22657 (Active promoters), 31340 (Enhancers), 2000 (Heterochromatin) and 32976 (Transcriptional elongation). Source data are provided as a Source Data file.

Supplementary Fig. 4: Histone exchange within active and bivalent gene promoters

a) Genome-wide comparison of H2B HA (log2) ChIP signals to the native H2B histone variant (log2). Reads are calculated within 5kb tiling windows (n=544480).

b) Correlation between H3.3 exchange and H2B-HA signal or RNA expression within 2kb windows surrounding TSSs of protein-coding genes (n=19592). Pearson correlation coefficients were calculated for all windows.

c-e) Heatmap representations of myc and HA ChIP-seq signals surrounding TSSs of protein-coding genes. Binning and sorting of genes was performed by decreasing myc levels of profiled histone variants – H3.3 (c), H3.1 (d) and H2B (e) respectively. Each row represent gene collections, and color indicate average ChIP-seq signals (log2). In summary, HA signals of TEV tagged variants were not influenced by myc sorting.

f) Boxplot representation of PRC2 proteins enrichment at the bivalent (n=3207) and non-bivalent (n=16385) gene groups. Black lines of boxplots indicate median, boxes correspond to first and third quartiles and whiskers represent maximum and minimum values of the distribution after removal of outliers – defined as points below 1st quartile or above 3rd quartile.

g) Heatmap showing Pearson correlation coefficients between three PRC2 components at the bivalent genes promoters (n=3207). Source data are provided as a Source Data file.

Supplementary Fig. 5: Histone occupancy around CTCF sites and modification patterns at asymmetrically exchanged CTCF sites

a) Metaprofiles and heatmap representations of H3.3 and H2B HA read counts surrounding CTCF sites (n=83810). Limits of heatmaps are set to 9.

b) Heatmap representation of histone marks within asymmetrically exchanged H3.1 and H2B variants. Source data are provided as a Source Data file.

Supplementary Fig. 6: Correlation plots between H3.3 occupancy and histone exchange at repetitive elements

a) Scatterplots between H3.3 fold enrichment over input and H3.3 (left) and H3.1 (right) exchange rates. Highlighted are repetitive elements with the highest enrichment of H3.3 between ref.[31] and this study. Source data are provided as a Source Data file.

Supplementary Fig. 7: Association between histone exchange levels and occupancy of canonical variants and generation of *HIRA*$^{-/-}$ cell lines

a, b) Histone exchange at regulatory sequences. Sorting of promoter sequences and enhancers by H3.1 HA (log2) (a) and H2B HA (log2) (b) intensity. Average myc and HA signal for all histone variants is then calculated per bin (25 bins in total).

c) Schematic representation of *HIRA* genomic region targeted with CRISPR/Cas9 gRNAs. Two gRNAs are designed to remove exon7 and introduce pre-mature stop codon. Lower: PCR validation of cell lines with biallelic deletion of exon7. Gels are representative of at least three independent experiments.

d) Sanger sequencing of *HIRA*$^{-/-}$ lines that are used for ChIP. Source data are provided as a Source Data file. Source data are provided as a Source Data file.

Supplementary Fig. 8: Implementation of sensor system *in vivo*

a) Mean myc and HA signals (log2) of H3.3 and H3.1 variants within 2kb windows surrounding TSSs (n=18029) of hepatocytes isolated from adult mice. Data are represented as mean ± SEM.

b) Barplot representation of H3.3 and H3.1 mean exchange (myc/HA, log2) over H3K27ac peaks (n=1141) of hepatocytes. Data are represented as mean ± SEM.

c) H3.1 mean exchange (myc/HA, log2) over H3K27ac peaks (n=16641) in MEFs. Error bars denoting SEM. Source data are provided as a Source Data file.

Supplementary Table 1

Cloning primers	
PRIMER1	CTG AAT CCT GCA GGA TGG CCC GAA CCA AGC AGA C
PRIMER2	CAT GTA CAA TTG AGC TCT CTC CCC CCG TAT CCG
PRIMER3	ATG CTG CAA TTG GGA GGT AGT GGC GGG GGA A
PRIMER4	ACT ACG GAA TTC CTA CAA GTC CTC CTC ACT AAT CAA TTT TTG
PRIMER5	CTG AAT ACC GGT CCT GCA GGA TCT CAC TTT TCC CTA CGG TTA CTT GCC A
PRIMER6	CAT GTA CAA TTG AGC CCT CTC CCC GCG GAT GC
PRIMER7	TCGTACCCTGCAGGATGCCAGAGCCTTCTAAGTCT
PRIMER8	GATCGAGGCCGGCCCTTGGAGCTGGTGTACTTGGT
PRIMER9	TCGATCGGCCGGCCGGAGGTAGTGGCGGGGGAA
PRIMER10	CATGCTGGATCCATCTCACTTTTCCCTACGGTTACTTGCCA
PRIMER11	TGCATGGCTAGCAGCCCTCTCCCCGCGGATG
PRIMER12	GGCCTCCAAGTCTTGACAGT
PRIMER13	GATATCCTTACGGAATACCACTTGCCACCTATCACC
PRIMER14	TTGCCTTTGTTACCTGTTCC
HIRA primers	
HIRA_cr_ex7_1_F	CAC CGG CCG ATT GAA TGG ACC CGA
HIRA_cr_ex7_1_R	AAA CTC GGG TCC ATT CAA TCG GCC
HIRA_cr_ex7_2_F	CAC CGC CCC TAA ACT GCA TGC CGC A
HIRA_cr_ex7_2_R	AAA CTG CGG CAT GCA GTT TAG GGG C
HIRA_cr_ex4_F	CAC CGT GTG TGC GGT GGT CAA ACA G
HIRA_cr_ex4_R	AAA CCT GTT TGA CCA CCG CAC ACA C
HIRA ex7 external F	GCCTTGGTGGAGGTGAAATA
HIRA ex7 external R	AACGGAGGAGGATATGAGAGAG
HIRA ex7 internal F	CTGAGAGGTCATTCTGGCTTAG
qPCR primers	
GAPDH Forward	AGGTCGGTGTGAACGGATTTG
GAPDH Reverse	TGTAGACCATGTAGTTGAGGTCA
β-Actin Forward	GGCTGTATTCCCCTCCATCG
β-Actin Reverse	CCAGTTGGTAACAATGCCATGT
Foxa2 Forward	CCCTACGCCAACATGAACTCG
Foxa2 Reverse	GTTCTGCCGGTAGAAAGGGA
Sox17 Forward	GATGCGGGATACGCCAGTG
Sox17 Reverse	CCACCACCTCGCCTTTCAC
Eomes Forward	GGCCCCTATGGCTCAAATTCC
Eomes Reverse	CCTGCCCTGTTTGGTGATG
Oct4 Forward	CGGAAGAGAAAGCGAACTAGC
Oct4 Reverse	ATTGGCGATGTGAGTGATCTG
H3.3A Forward	GTGTCCTTACCATGGCTCGT
H3.3A Reverse	CAGGCCTGTAACGATGAGGT
H3.3B Forward	GCCTCGGTCTCAGCAAGG
H3.3B Reverse	CTTGGTGGCCAGCTGTTT

H2B Forward	GCCGCAAGGAGAGCTA
H2B Reverse	CGCTTGTTGTAATGCGCCAG
H3.1 Forward	TCG TAC TAA GCA GAC CGC TC
H3.1 Reverse	TGG ATG TCC TTG GGC ATG AT
H3.3-HA Forward	AGTTGGCTCGCCGGATAC
H3.3-HA Reverse	GCATAGTCCGGGACGTCATA
TEV Forward	ACC ACG ACT TTG CAA CAA CA
TEV Reverse	CGT TCT TCC CTT TGT GGC TC
H3.1-HA Forward	CCAAGCGTGTCACCATCAT
H3.1-HA Reverse	CGTAATCCGGAACGTCGTAA
H2B-HA Forward	TCA CCA AGT ACA CCA GCT CC
H2B-HA Reverse	GCATAGTCCGGGACGTCATA

Supplementary Table 2

File	Total number of reads	Number of reads after alignign and filtering	Sequencing type	R1 length	R2 length
H2B_cleavable_I_HA	123218597	78996254	paired-end	51	51
H2B_cleavable_I_myc	102988897	72892105	paired-end	51	51
H2B_cleavable_II_HA	143269409	96336936	paired-end	51	51
H2B_cleavable_II_myc	66569707	53569534	paired-end	51	51
H2B_NC_I_HA	45781295	27903389	paired-end	51	51
H2B_NC_I_myc	47755948	28906325	paired-end	51	51
H3.1_cleavable_I_HA	107717654	62935026	paired-end	92	30
H3.1_cleavable_I_myc	100983979	61158192	paired-end	92	30
H3.1_cleavable_II_HA	100427340	60248395	paired-end	61	61
H3.1_cleavable_II_myc	62751701	40743057	paired-end	61	61
H3.1_cleavable_HIR_KO_1_I_HA	69658835	42387537	paired-end	61	60
H3.1_cleavable_HIR_KO_1_I_myc	74873544	48155315	paired-end	61	60
H3.1_cleavable_HIR_KO_1_II_HA	82520940	59511044	paired-end	86	36
H3.1_cleavable_HIR_KO_1_II_myc	115562906	77406838	paired-end	86	36
H3.1_cleavable_HIR_KO_2_I_HA	49598949	35839244	paired-end	61	61
H3.1_cleavable_HIR_KO_2_I_myc	69338197	53008939	paired-end	61	61
H3.1_cleavable_HIR_KO_2_II_HA	42835327	29900606	paired-end	86	36
H3.1_cleavable_HIR_KO_2_II_myc	38277278	17424709	paired-end	86	36
H3.1_NC_I_HA	35149552	22777825	paired-end	51	51
H3.1_NC_I_myc	33415140	21330145	paired-end	69	15
H3.3_cleavable_I_HA	48795842	32119551	paired-end	51	51
H3.3_cleavable_I_myc	35106749	23598480	paired-end	51	51
H3.3_cleavable_II_HA	39471531	26138179	paired-end	51	51
H3.3_cleavable_II_myc	28603328	19387559	paired-end	51	51
H3.3_cleavable_HIR_KO_1_I_HA	47738224	28745005	paired-end	61	60
H3.3_cleavable_HIR_KO_1_I_myc	37749938	25770895	paired-end	61	60
H3.3_cleavable_HIR_KO_2_I_HA	94675809	54527416	paired-end	61	61
H3.3_cleavable_HIR_KO_2_I_myc	93396093	61161402	paired-end	61	61
H3.3_NC_I_HA	38294136	24709323	paired-end	90	32
H3.3_NC_I_myc	38110362	24353921	paired-end	51	51
Hepatocytes H3.3 HA	37230876	28624752	paired-end	61	61
Hepatocytes H3.3 myc	41861299	30311017	paired-end	61	61
Hepatocytes H3.1 HA	33009943	23350128	paired-end	61	61
Hepatocytes H3.1 myc	17501043	12542897	paired-end	61	61
Hepatocytes H3.3 K27ac	38815717	20710217	paired-end	61	61
Hepatocytes H3.1 K27ac	37329219	24590774	paired-end	61	61
MEF H3.1 HA	36456339	27348202	paired-end	86	36
MEF H3.1 myc	18114567	8683908	paired-end	86	36
MEF H3.1 K27ac	30779942	13727142	paired-end	86	36
H2B_cleavable_H2B	120279444	77886263	paired-end	51	51

Uncropped blot of Supplementary Fig. 1c

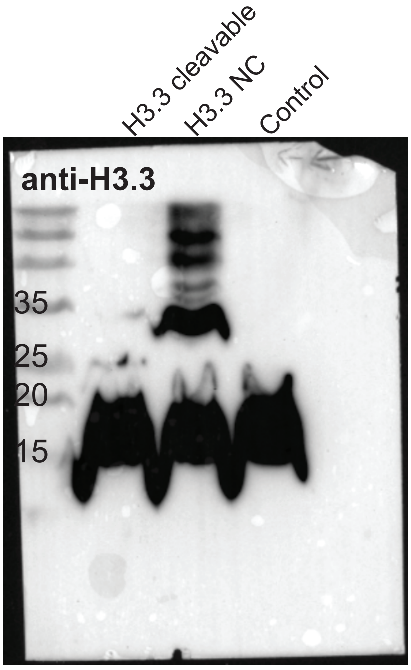

Uncropped gel of Supplementary Fig. 7c

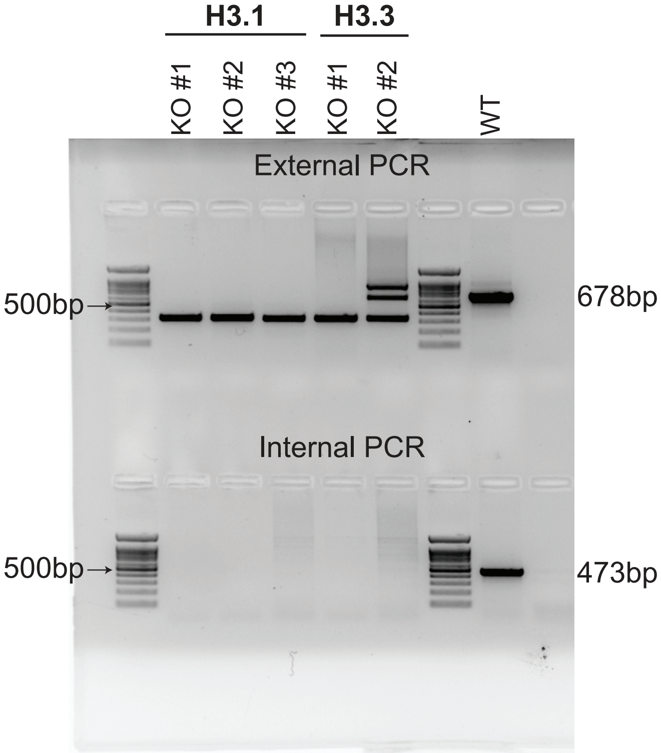

Supplement: Supplementary file 1 — Supplementary information [file 41467_2023_39477_MOESM1_ESM.pdf]
