# Supplementary material for: Histone exchange sensors reveal variant specific dynamics in mouse embryonic stem cells
Source: Nat Commun. 2023 Jun 26;14:3791. doi: 10.1038/s41467-023-39477-3 (PMC10293259; doi:10.1038/s41467-023-39477-3)
Supplement: Supplementary file 2 — Reporting summary [file 41467_2023_39477_MOESM2_ESM.pdf]

nature portfolio

Corresponding author(s): Yonatan Stelzer

Last updated by author(s): Jun 5, 2023

Reporting Summary

Nature Portfolio wishes to improve the reproducibility of the work that we publish. This form provides structure for consistency and transparency in reporting. For further information on Nature Portfolio policies, see our Editorial Policies and the Editorial Policy Checklist.

Statistics

For all statistical analyses, confirm that the following items are present in the figure legend, table legend, main text, or Methods section.

n/a	Confirmed	
☐	☒	The exact sample size (*n*) for each experimental group/condition, given as a discrete number and unit of measurement
☐	☒	A statement on whether measurements were taken from distinct samples or whether the same sample was measured repeatedly
☐	☒	The statistical test(s) used AND whether they are one- or two-sided<br>*Only common tests should be described solely by name; describe more complex techniques in the Methods section.*
☒	☐	A description of all covariates tested
☐	☒	A description of any assumptions or corrections, such as tests of normality and adjustment for multiple comparisons
☐	☒	A full description of the statistical parameters including central tendency (e.g. means) or other basic estimates (e.g. regression coefficient) AND variation (e.g. standard deviation) or associated estimates of uncertainty (e.g. confidence intervals)
☐	☒	For null hypothesis testing, the test statistic (e.g. *F*, *t*, *r*) with confidence intervals, effect sizes, degrees of freedom and *P* value noted<br>*Give P values as exact values whenever suitable.*
☒	☐	For Bayesian analysis, information on the choice of priors and Markov chain Monte Carlo settings
☒	☐	For hierarchical and complex designs, identification of the appropriate level for tests and full reporting of outcomes
☐	☒	Estimates of effect sizes (e.g. Cohen's *d*, Pearson's *r*), indicating how they were calculated

Our web collection on statistics for biologists contains articles on many of the points above.

Software and code

Policy information about availability of computer code

Data collection	Tools, softwares and R packages used in this study: bcl2fastq (Illumina) (version 2.17.1.14), bowtie2 (version 2.3.0), MACS2 (version 2.2.7.1), samtools (version 1.15.1), GenomicRanges (version 1.38.0), Rsamtools (version 2.2.3), rtracklayer (version 1.46.0), GenomicAlignments (version 1.22.1), AnnotationHub (version 2.18.0), TxDb.Mmusculus.UCSC.mm10.knownGene (version 3.10.0), genomation (version '1.18.0')
Data analysis	Custom codes used for generating figures are available upon request.

For manuscripts utilizing custom algorithms or software that are central to the research but not yet described in published literature, software must be made available to editors and reviewers. We strongly encourage code deposition in a community repository (e.g. GitHub). See the Nature Portfolio guidelines for submitting code & software for further information.

Data

Policy information about availability of data

All manuscripts must include a data availability statement. This statement should provide the following information, where applicable:

- Accession codes, unique identifiers, or web links for publicly available datasets
- A description of any restrictions on data availability
- For clinical datasets or third party data, please ensure that the statement adheres to our policy

The ChIP-seq data generated in this study have been deposited in NCBI's Gene Expression Omnibus (Edgar et al, 2002) under accession number GSE213076 (https://www.ncbi.nlm.nih.gov/geo/query/acc.cgi?acc=GSE213076). Previously published ChIP-seq data that were used for analysis are available under the following accession numbers: GSM723017, GSM723020, GSM747539, GSM747540, GSM747541, GSM1891651, GSM1891652, GSM801982, GSM801983, GSM747542,

GSM747547, GSM747548, GSM1526289, GSM1526285, GSM2533855, GSM1199188, GSM1199182, GSM491760, GSM747545, GSM747546, GSM1375155, GSM1251941, GSM1555116, GSM1429923, GSM3058311, GSM3058312, GSM3058339, GSM3058340,GSM6597072,GSM6597078, GSM769029, GSM769030.
GRCm38.p6 genome assembly. Annotation of genomic regions was taken from: https://github.com/guifengwei/ChromHMM_mESC_mm10. RNA gene expression of mESC and MEFs was taken from: GSE89210 and GSE153578, respectively. Additionally, expression levels of hepatocytes were defined in "Richter, M. L. et al. Single-nucleus RNA-seq2 reveals functional crosstalk between liver zonation and ploidy". Methylation data in mESC was taken from: GSE30206. Coordinates of A and B were defined in "Bonev, B. et al. Multiscale 3D Genome Rewiring during Mouse Neural Development. Cell 171, 557-572.e24 (2017)". HIRA peaks were defined in "Xiong, C. et al. UBN1/2 of HIRA complex is responsible for recognition and deposition of H3.3 at cis-regulatory elements of genes in mouse ES cells. BMC Biol. 16, 110 (2018)". The chain file used for liftOver conversion was downloaded from
http://hgdownload.soe.ucsc.edu/goldenPath/mm9/liftOver/mm9ToMm10.over.chain.gz. Murine repeat sequences were downloaded from Repbase (http://www.girinst.org/repbase/). Coordinates of full-length IAP elements were downloaded from https://github.com/elsasserlab/publicchip
CTCF-bound regions, and their motif orientation was defined in "Chang, L.-H. et al. A complex CTCF binding code defines TAD boundary structure and function. Preprint at https://doi.org/10.1101/2021.04.15.440007 (2021) doi:10.1101/2021.04.15.440007". CTCF motif sites were downloaded from AnnotationHub R package, using the following code:

```
ah <- AnnotationHub()
query_data <- subset(ah, preparerclass == "CTCF")
subset(query_data, species == "Mus musculus" & genome == "mm10" & dataprovider == "JASPAR 2022")
CTCF_mm10_all <- query_data[["AH95568"]]
```

Research involving human participants, their data, or biological material

Policy information about studies with human participants or human data. See also policy information about sex, gender (identity/presentation), and sexual orientation and race, ethnicity and racism.

Reporting on sex and gender	not applicable
Reporting on race, ethnicity, or other socially relevant groupings	not applicable
Population characteristics	not applicable
Recruitment	not applicable
Ethics oversight	not applicable

Note that full information on the approval of the study protocol must also be provided in the manuscript.

Field-specific reporting

Please select the one below that is the best fit for your research. If you are not sure, read the appropriate sections before making your selection.

[x] Life sciences [ ] Behavioural & social sciences [ ] Ecological, evolutionary & environmental sciences

For a reference copy of the document with all sections, see nature.com/documents/nr-reporting-summary-flat.pdf

Life sciences study design

All studies must disclose on these points even when the disclosure is negative.

Sample size	No sample size calculation was performed. ChIP-seq experiments on cell lines expressing cleavable form of exchange sensor have at least two replicates, in line with a common practice in the genomic field. ChIP-seq of non-cleavable version was representing a control experiment used mostly to validate specificity of anti-myc and anti-HA antibodies, and therefore one sample per group was sufficient for the analysis. Additionally, one sample was used for profiling native H2B signal in cells expressing cleavable sensor, as a control to distribution of tagged H2B variant. Similarly, only one sample derived from in vivo tissues were used for sequencing, as intention was to demonstrate utility of the system for in vivo application, and therefore one sample was sufficient. Exception to this was H3K27ac that was profiled on two hepatocytes samples, isolated from either H3.1- or H3.3-sensor carrying mice. RT-qPCR experiments were performed in three or four technical replicates. More technical replicates were not needed for RT-qPCR as we did not see big variation between samples.
Data exclusions	Sequencing libraries that did not match quality-control measures, showing low internal correlations to respective repeats were not considered for the analysis. ChIP-sequencing for H3.1-cleavable sensor was performed three times, whereby one of the replicate was excluded from the analysis, due to lower correlation of HA signal to HA signal of other two replicates.
Replication	ChIP-seq experiments were performed in two replicates. Genome-studies were performed on individual replicates to verify conclusions for each figure, but for the final versions of figures replicates were pooled together. In case when one of replicates was sequenced to a higher depth, pooling was performed after down-sampling of a replicate with higher coverage prior to pooling such that it matches sequencing depth of less sequenced replicate. ChIP-seq experiments involving HIRA knock-out were performed on two independent cell lines (for both, H3.1-tagged and H3.3-tagged samples) with different HIRA genotypes. Western blot experiments demonstrating expression and cleavage kinetics of TEV enzyme are performed in at least three independent experiments with the same results.
Randomization	Randomization was not relevant for cell-culture based study. Comparisons were performed between WT and KO cell lines. Mice were allocated into experimental groups according to their genotype.

Blinding	Blinding was not relevant for the study, since group allocation was performed

Reporting for specific materials, systems and methods

We require information from authors about some types of materials, experimental systems and methods used in many studies. Here, indicate whether each material, system or method listed is relevant to your study. If you are not sure if a list item applies to your research, read the appropriate section before selecting a response.

Materials & experimental systems

n/a	Involved in the study
☐	☒ Antibodies
☐	☒ Eukaryotic cell lines
☒	☐ Palaeontology and archaeology
☐	☒ Animals and other organisms
☒	☐ Clinical data
☒	☐ Dual use research of concern
☒	☐ Plants

Methods

n/a	Involved in the study
☐	☒ ChIP-seq
☒	☐ Flow cytometry
☒	☐ MRI-based neuroimaging

Antibodies

Antibodies used

anti-HA (12CA5) and anti-myc (9E10) antibodies were supernatant of the respective hybridoma cell cultures grown in miniPERM bioreactors in-house by the Weizmann Institute Core Facility Antibody Unit. About 5ug of anti-HA and anti-myc antibodies were used for ChIP. For Western blot, anti-HA antibody was added in 1:1000 dilution.
anti-α-GAPDH antibody [EPR16891] (Abcam, Cat #ab181602; RRID: AB_2630358, used in 1:5000 dilution)
anti-α-tubulin antibody (Millipore, Cat#ABT170, used in 1:2000 dilution)
anti-HIRA antibody (Active Motif, clone WC119.2H11, used in 1:1000 dilution)
anti-H3.3 (Abcam, ab176840) added in 1:1000
anti-H3 (Abcam, ab1791) added in 1:5000
Goat anti-Rabbit IgG (H+L), HRP (Invitrogen, 31460) added in 1:10000
Goat anti-Mouse IgG (H+L), HRP (Invitrogen, 31430) added in 1:10000
anti-H2B (Abcam, ab1790) ~3ug for ChIP assay.
anti-H3K27ac (Abcam, ab4729) ~3ug for ChIP assays.

Validation

anti-HA (12CA5) and anti-myc (9E10) antibodies were validated by Western blots (Fig. 1c and Fig. 2b) and by ChIP experiments in cells bearing non-cleavable form of exchange sensor, resulting in high correlation between myc and HA antibodies (Fig. 1d and Supplementary Fig. 3d).
Anti-α-GAPDH antibody (ab181602) from Abcam website: https://www.abcam.com/products/primary-antibodies/gapdh-antibody-epr16891-loading-control-ab181602.html
Anti-α-tubulin antibody (ABT170) from Millipore website: https://www.merckmillipore.com/INTL/en/product/Anti-alpha-Tubulin-Antibody-nontyrosinated,MM_NF-ABT170?ReferrerURL=https%3A%2F%2Fwww.google.com%2F
Anti-HIRA antibody (WC119.2H11) from Active Motif website: https://www.activemotif.com/catalog/details/39557
Anti-H3.3 antibody (ab176840) from Abcam website: https://www.abcam.com/products/primary-antibodies/histone-h33-antibody-epr17899-chip-grade-ab176840.html
Anti-H3 antibody (ab1791) from Abcam website: https://www.abcam.com/products/primary-antibodies/histone-h3-antibody-nuclear-marker-and-chip-grade-ab1791.html
Anti-H2B antibody (ab1790) from Abcam website: https://www.abcam.com/products/primary-antibodies/histone-h2b-antibody-chip-grade-ab1790.html
Anti-H3K27ac antibody (ab4729) from Abcam website: https://www.abcam.com/products/primary-antibodies/histone-h3-acetyl-k27-antibody-chip-grade-ab4729.html
Goat anti-Rabbit IgG (H+L), HRP (Invitrogen, 31460): https://www.thermofisher.com/antibody/product/Goat-anti-Rabbit-IgG-H-L-Secondary-Antibody-Polyclonal/31460
Goat anti-Mouse IgG (H+L), HRP (Invitrogen, 31430): https://www.thermofisher.com/antibody/product/Goat-anti-Mouse-IgG-H-L-Secondary-Antibody-Polyclonal/31430

Eukaryotic cell lines

Policy information about cell lines and Sex and Gender in Research

Cell line source(s)	Mouse embryonic stem cells expressing cleavable or non-cleavable forms of the reporter system are generated by site-specific integration into H11 locus of a male-derived (XY) V6.5 mouse embryonic stem cells (Jaenisch lab, MIT. RRID:CVCL_C865) carrying three partial attP sites and generated previously in-house. HIRA KO cell lines are subsequently generated on the background of cells carrying either H3.1- or H3.3-reporter system.
Authentication	Karyotyping, PCR and Sanger Sequencing. HIRA KO cell lines were also validated at the protein levels by Western blot.

Mycoplasma contamination	Cells were routinely tested for mycoplasma and no mycoplasma contamination was detected.
Commonly misidentified lines (See ICLAC register)	None of commonly misidentified cell lines were used.

Animals and other research organisms

Policy information about studies involving animals; ARRIVE guidelines recommended for reporting animal research, and Sex and Gender in Research

Laboratory animals	To generate chimeric mice, mouse embryonic stem cells bearing cleavable version of H3.3-sensor or H3.1-sensor were injected into (C57BL/6xDBA) B6D2F1 host blastocyst (Envigo), harvested after hormon priming of 3-4-weeks old B6D2F1 females by intraperitoneal injection of pregnant mare serum gonadotropin (PMSG, Vetmarket) and followed by an injection of human chorionic gonadotropin (hCG, Sigma) 46 hr later.<br>For germline transmission, male chimera mice were mated with C57BL/6 females and progeny was genotyped for transgenic alleles by PCR. Male and female offspring carrying the sensor allele were further bred to obtain homozygous mice.<br>The following sentence was added to the manuscript: "All animals are given free access to food and water and were maintained under controlled conditions with 12 hr light–dark cycle at 22 °C degrees (±2 °C) and 55% humidity (±10%). Breeding experiments were performed on mice that were 8-12 weeks old." For isolation of hepatocytes 14-weeks old male mice carrying either H3.1- or H3.3-sensor were sacrificed.
Wild animals	No wild animals were used.
Reporting on sex	Sex was determent upon weaning.
Field-collected samples	No field-collected samples were used.
Ethics oversight	Mice were handled in accordance with Animal Protection Guidelines of Weizmann Institute of Science, Rehovot, Israel. Animal experiments were approved by relevant Weizmann Institute IACUC (#08241020-2 and #02610320-2).

Note that full information on the approval of the study protocol must also be provided in the manuscript.

Plants

Seed stocks	not applicable
Novel plant genotypes	not applicable
Authentication	not applicable

ChIP-seq

Data deposition

- [x] Confirm that both raw and final processed data have been deposited in a public database such as GEO.
- [x] Confirm that you have deposited or provided access to graph files (e.g. BED files) for the called peaks.

Data access links<br>*May remain private before publication.*	GSE213076 (https://www.ncbi.nlm.nih.gov/geo/query/acc.cgi?acc=GSE213076)
Files in database submission	H2B_NC_I_myc.bw;<br>H2B_NC_I_HA.bw;<br>H2B_cleavable_I_HA.bw;<br>H2B_cleavable_I_myc.bw;<br>H2B_cleavable_II_HA.bw;<br>H2B_cleavable_II_myc.bw;<br>H3.l_NC_I_HA.bw;<br>H3.l_NC_I_myc.bw;<br>H3.l_cleavable_I_HA.bw;<br>H3.l_cleavable_I_myc.bw;<br>H3.l_cleavable_II_HA.bw;<br>H3.l_cleavable_II_myc.bw;<br>H3.l_cleavable_HIR_KO_l_I_HA.bw;<br>H3.l_cleavable_HIR_KO_l_I_myc.bw;<br>H3.l_cleavable_HIR_KO_l_II_HA.bw;<br>H3.l_cleavable_HIR_KO_l_II_myc.bw;<br>H3.l_cleavable_HIR_K0_2_1_HA.bw;<br>H3.l_cleavable_HIR_K0_2_1_myc.bw;<br>H3.l_cleavable_HIR_K0_2_11_HA.bw;

H3.l_cleavable_HIR_K0_2_11_myc.bw;
H3.3_NC_I_HA.bw;
H3.3_NC_l_myc.bw;
H3.3_cleavable_l_HA.bw;
H3.3_cleavable_l_myc.bw;
H3.3_cleavable_ll_HA.bw;
H3.3_cleavable_ll_myc.bw;
H3.3_cleavable_HIR_KO_l_l_HA.bw;
H3.3_cleavable_HIR_KO_l_l_myc.bw;
H3.3_cleavable_HIR_K0_2_1_HA.bw;
H3.3_cleavable_HIR_K0_2_1_myc.bw
H2B_FAST_H2B_l.bw
Hepatocytes_H3.1_HA.bw
Hepatocytes_H3.1_K27ac.bw
Hepatocytes_H3. 1_myc.bw
Hepatocytes_H3.3_HA.bw
Hepatocytes_H3.3_K27ac.bw
Hepatocytes_H3.3_myc.bw
MEF_H3.1_HA.bw
MEF_H3.1_K27ac.bw
MEF_H3.1_myc.bw

Genome browser session
(e.g. UCSC)

no longer applicable

Methodology

Replicates

For cells expressing cleavable form of exchange sensor at least two replicates were processed. HIRA experiments were performed no two different clones of a different deletion genotypes. H3K27ac levels of hepatocytes was performed on two samples derived from H3.1-sensor and H3.3-sensor mice, separately.

Sequencing depth

File Total number of reads Number of reads after aligning and filtering Sequencing type Rl length R2 length
H2B_cleavable_l_HA 123218597 78996254 paired-end 51 51
H2B_cleavable_l_myc 102988897 72892105 paired-end 51 51
H2B_cleavable_ll_HA 143269409 96336936 paired-end 51 51
H2B_cleavable_ll_myc 66569707 53569534 paired-end 51 51
H2B_NC_I_HA 45781295 27903389 paired-end 51 51
H2B_NC_l_myc 47755948 28906325 paired-end 51 51
H3.1_cleavable_l_HA 107717654 62935026 paired-end 92 30
H3.1_cleavable_l_myc 100983979 61158192 paired-end 92 30
H3.1_cleavable_ll_HA 100427340 60248395 paired-end 61 61
H3.1_cleavable_ll_myc 6275170140743057 paired-end 61 61
H3.1_cleavable_HIR_K0_1_1_HA 69658835 42387537 paired-end 61 60
H3.1_cleavable_HIR_K0_1_1_myc 74873544 48155315 paired-end 61 60
H3.1_cleavable_HIR_KO_l_ll_HA 82520940 59511044 paired-end 86 36
H3.1_cleavable_HIR_KO_l_ll_myc 115562906 77406838 paired-end 86 36
H3.1_cleavable_HIR_K0_2_1_HA 49598949 35839244 paired-end 61 61
H3.1_cleavable_HIR_K0_2_1_myc 69338197 53008939 paired-end 61 61
H3.1_cleavable_HIR_K0_2_11_HA 42835327 29900606 paired-end 86 36
H3.1_cleavable_HIR_K0_2_11_myc 3827727817424709 paired-end 86 36
H3.1_NC_I_HA 35149552 22777825 paired-end 51 51
H3.1_NC_l_myc 33415140 21330145 paired-end 69 15
H3.3_cleavable_l_HA 48795842 32119551 paired-end 51 51
H3.3_cleavable_l_myc 35106749 23598480 paired-end 51 51
H3.3_cleavable_ll_HA 3947153126138179 paired-end 51 51
H3.3_cleavable_ll_myc 2860332819387559 paired-end 51 51
H3.3_cleavable_HIR_K0_1_1_HA 47738224 28745005 paired-end 61 60
H3.3_cleavable_HIR_K0_1_1_myc 37749938 25770895 paired-end 61 60
H3.3_cleavable_HIR_K0_2_1_HA 94675809 54527416 paired-end 61 61
H3.3_cleavable_HIR_K0_2_1_myc 93396093 61161402 paired-end 61 61
H3.3_NC_I_HA 38294136 24709323 paired-end 90 32
H3.3_NC_l_myc 38110362 24353921 paired-end 51 51
Hepatocytes H3.3 HA 37230876 28624752 paired-end 61 61
Hepatocytes H3.3 myc 41861299 30311017 paired-end 61 61
Hepatocytes H3.1 HA 33009943 23350128 paired-end 61 61
Hepatocytes H3.1 myc 1750104312542897 paired-end 61 61
Hepatocytes H3.3 K27ac 38815717 20710217 paired-end 61 61
Hepatocytes H3.1 K27ac 37329219 24590774 paired-end 61 61
MEF H3.1 HA 36456339 27348202 paired-end 86 36
MEF H3.1 myc 18114567 8683908 paired-end 86 36

	MEF H3.1 K27ac 30779942 13727142 paired-end 86 36<br>H2B_cleavable_H2B 120279444 77886263 paired-end 51 51
Antibodies	Anti-HA (12CA5)<br>Anti-myc (9E10)<br>H2B (Abcam, ab1790)<br>anti-H3K27ac (Abcam, ab4729)
Peak calling parameters	Alignment of paired-end reads was performed as: bowtie2 -p 16 -x $PATH_TO_INDEXED_FILES -1 $sample_name_R1.fastq.gz -2 $sample_name_R2.fastq.gz |samtools sort -o $sample_name.bam<br>Alignment of single reads was performed as: bowtie2 -p 16 -x $PATH_TO_INDEXED_FILES -U $sample_name.fastq.gz |samtools sort -o $sample_name.bam<br>Calling peaks on H3K27ac used in Fig. 1k: macs2 callpeak-t $sample_name.bam -c H3K27ac_input.bam -g 1.87e9 -n H3K27ac -f BAM --nolambda --outdir $dir_name Accession number of H3K27ac for Fig. 1k: GSM1891651, GSM1891652 and input files: GSM747545, GSM747546<br>Calling peaks on H3K27ac used in Supplementary Fig. 8b-c: macs2 callpeak -t $sample_name.bam -g 1.87e9 -n $sample_name -f BAM --nolambda --outdir $dir_name
Data quality	Correlation of myc and HA tags between biological repeats were calculated prior to pooling of sequences. For Supplementary Fig. 8b H3K27ac peaks common between the two datasets were used for the analysis.
Software	bcl2fastq (Illumina) (version 2.17.1.14), bowtie2 (version 2.3.0), MACS2 (version 2.2.7.1), samtools (version 1.15.1), GenomicRanges (version 1.38.0), Rsamtools (version 2.2.3), rtracklayer (version 1.46.0), GenomicAlignments (version 1.22.1), AnnotationHub (version 2.18.0), TxDb.Mmusculus.UCSC.mm10.knownGene (version 3.10.0), genomation (version ‘1.18.0’)
